# Utilization of Yeast Cells as Alternative Carriers in the Microencapsulation of Black Chokeberry (*Aronia melanocarpa*) Phenolic Extract

**DOI:** 10.3390/foods14040625

**Published:** 2025-02-13

**Authors:** Özlem Aktürk Gümüşay, İnci Cerit, Omca Demirkol

**Affiliations:** 1 Department of Gastronomy and Culinary Arts, Maltepe University, Maltepe, 34857 İstanbul, Turkey; ozlemgumusay@maltepe.edu.tr; 2 Department of Food Engineering, Sakarya University, Esentepe, 54187 Sakarya, Turkey; incicantik@sakarya.edu.tr

**Keywords:** aronia, encapsulation, freeze-drying, *Saccharomyces cerevisiae*, polyphenols, in vitro digestion

## Abstract

The structure of yeast cells, which are rich in bioactive compounds, makes them an attractive encapsulation vehicle due to their antioxidant, antibacterial, and antimutagenic properties. In this study, black chokeberry extract was encapsulated with different wall materials (maltodextrin, gum arabic, mixture of maltodextrin and gum arabic, plasmolyzed yeast, and non-plasmolyzed yeast) by freeze-drying. While the highest encapsulation efficiency was obtained with maltodextrin (98.82%), non-plasmolyzed yeast (86.58%) emerged as a viable alternative to gum arabic. The largest particle size was observed in plasmolyzed yeast microcapsules. Yeast-coated capsules exhibited a spheroidal morphology. Differential Scanning Calorimetry revealed high thermal stability for all microcapsules, with the gum arabic-coated microcapsules demonstrating the greatest stability. After the simulated gastric and intestinal fluid treatment, plasmolyzed yeast provided the highest retention, with 63.45% and 77.55% of phenolics, respectively. The highest 2,2-Diphenyl-1-picrylhydrazyl (DPPH) activities were found in yeast microcapsules, with no significant difference between them. In 2,2′-Azino-bis (3-ethylbenzothiazoline-6-sulfonic acid) (ABTS^•+^) scavenging activity, the least loss (approximately 10%) was observed in non-plasmolyzed yeast samples after intestinal digestion. These results showed that yeast can be used as an alternative coating material in the encapsulation of phenolics, and it contributes to the bioavailability of microcapsules with its protective effect during digestion.

## 1. Introduction

Bioactive compounds, including phenolic substances found in black chokeberries (*Aronia melanocarpa* (Michaux) Elliot), play a crucial role in reducing oxidative stress and have potential health benefits, such as preventing chronic diseases; however, their stability is compromised by exposure to light, oxygen, and moisture, requiring protective measures for effective utilization [1,2,3,4]. Microencapsulation techniques are one of the most widely used methods to protect the beneficial properties of phenolics from degradation due to an oxidizing environment [5]. Microencapsulation offers various benefits such as protection of sensitive materials from environmental conditions, enhancement of stability, controlled release of active ingredients, and overcoming the harmful effects of the gastrointestinal system. In addition, it masks the astringent taste often found in phytochemicals, mainly in polyphenols [6,7]. There have been several encapsulation techniques, including freeze-drying, spray-drying, coacervation, fluid bed coating, and emulsion/solvent evaporation. The common purpose of all these methods is dispersing the core material within the wall material and forming microcapsules of the desired size and shape [8]. Among these techniques, freeze-drying provides coating of core material along with a protective matrix at very low temperatures and under reduced pressure. Freeze-drying is a simple and ideal encapsulation method due to its low temperature and application in preserving heat-sensitive materials [9,10].

The choice of coating material is another crucial factor for the efficiency of encapsulation and stability of capsules. There are different types of coating agents, including carbohydrate-based (starch, gum arabic, maltodextrin, alginate), lipid-based (stearic acid, waxes), and protein-based (whey protein, gelatin, casein) materials [11]. Among these numerous carriers, maltodextrin is the most widely preferred polysaccharide coating material due to its several functions, such as low viscosity, high water solubility, and low cost [12]. Similarly to maltodextrin, gum arabic is also popular due to its high solubility in water and film-forming properties. Combinations of maltodextrin and gum arabic have been used to encapsulate phenolics extracted from black chokeberries [13,14,15]. In recent years, yeasts have also started to be used as a wall material in encapsulation. Both hydrophilic and hydrophobic core materials can be encapsulated in yeast cells such as β-carotene, limonene, or anthocyanins [16,17,18]. Compared to conventional wall materials such as maltodextrin or gum arabic, yeast cells provide enhanced stability for bioactive compounds [19]. Moreover, yeast itself as wall material can contribute nutrients such as proteins, B vitamins, β-glucans, while conventional wall materials are typically inert and cannot add any biological activity to the product [20,21,22].

In encapsulation processes involving yeasts, the effect of plasmolysis varies by core material, with some studies reporting increased loading capacity [23], while others show reduced efficiency [24,25]. Optimizing the conditions to maximize the loading capacity of the target compound is crucial for achieving desired outcomes. To the best of the authors’ knowledge, only one study has examined the encapsulation of black chokeberry extract using yeast cells. In that study, Kurek et al. (2023) focused on the physical properties of the yeast capsules, but did not investigate in vitro digestion [26]. The aim of this study was to evaluate the encapsulation of phenolic compounds from black chokeberry using both plasmolyzed and non-plasmolyzed *Saccharomyces cerevisiae* cell walls, as well as maltodextrin and gum arabic, and to compare the encapsulation efficiency through morphological, conformational, and in vitro digestion analyses.

## 2. Materials and Methods

### 2.1. Materials

Maltodextrin (DE 10-12) and gum arabic were purchased from Alfasol Co. (Istanbul, Turkey). Baker’s yeast (Dr. Oetker, Bielefeld, Germany) was purchased from a local market in Sakarya (Turkey). All other solvents and chemicals were of analytical grade (Sigma Aldrich, St. Louis, MO, USA).

Fresh black chokeberry [*Aronia melanocarpa* (Michaux) Elliot] was obtained from Yalova in Turkey. Fresh black chokeberries were dried at 50 °C for 48 h, ground into powder and sieved through a sieve (70-mesh). The moisture content of dried chokeberry powder was 8.28%. The powder was stored at +4 °C until further extraction.

### 2.2. Extract Preparation

The powdered chokeberries were extracted with 50% ethanol using an ultrasonicator (Bandelin 140 Sonoplus HD2200, Berlin, Germany) that was equipped with a 4.5 mm diameter and 130 mm length probe in frequency mode (10 s active, rest 2 s). The extraction time was 61 min, the ultrasonic power was 120 W, and the solid-to-solvent ratio was 1:30. The temperature was kept constant at room temperature (25 °C) with an ice bath. These extraction conditions were previously chosen as optimal for the extractions of chokeberry polyphenols. The relevant study in which the extraction conditions were optimized has not been published yet.

### 2.3. Encapsulation of Chokeberry Extracts by Freeze-Drying Method

#### 2.3.1. Preparation of Maltodextrin and Gum Arabic Solutions

The crude extract obtained by ultrasonic extraction was concentrated using an evaporator until the total solid content reached 11%, and ethanol was removed. For the preparation of maltodextrin (MD) and gum arabic (GA) solutions, 150 g of each material was weighed separately and dissolved in 500 mL of distilled water at ambient temperature (25 ± 1 °C). Additionally, an MD+GA mixture was prepared by combining equal amounts of the MD and GA solutions. The solutions were stirred at 750 rpm for 3 h to achieve a total solids concentration of 30%. The solution was then stored in a refrigerator for 24 h to allow complete hydration.

#### 2.3.2. Preparation of Yeast Solutions

To prepare non-plasmolyzed yeast, baker’s yeast (50 g) was washed with phosphate buffer (500 mL, pH 6.8). The supernatant was then collected by centrifugation at 4100× *g* for 10 min. The washing process was repeated five times using distilled water. After washing, the cells were freeze-dried at −50 °C for 48 h using a lyophilizer (Labconco, 201 Kansas City, MO, USA). This group of yeast was classified as non-plasmolyzed yeast (NPY).

For the plasmolysis process, baker’s yeast (50 g) was mixed with 10% NaCl solution (500 mL) and kept in a shaking incubator at 55 °C at 120 rpm for 48 h. After centrifugation (4100× *g*, 10 min), yeast cells were washed five times and freeze-dried [25]. This group of yeast was categorized as plasmolyzed (PY). Plasmolyzed and non-plasmolyzed yeast were dissolved in distilled water (10%).

#### 2.3.3. Encapsulation Process

For the preparation of solutions containing black chokeberry extract and 5 different wall materials (MD, GA, MD+GA, PY, and NPY), 3 different mixing ratios were determined with preliminary experiments. The extract and each of the 5 wall materials were mixed separately in weight ratios (*w*/*w*) of 1:5, 1:15, and 1:25 (extract–wall material). In total, 15 different solutions were obtained. They were shaken at 140 rpm at 27 °C for 3 h. At the end of the incubation, the samples were centrifuged (13,130× *g*, 10 min), and the supernatant was discarded [27]. Samples were frozen to –18 °C in a refrigerator prior to freeze-drying. Then, the samples were encapsulated using a freeze-dryer at −50 °C for 48 h. Finally, the dried samples were ground with a blender (Waring, Tonrrington, CT, USA) and stored at –18 °C until analysis.

### 2.4. Encapsulating Efficiency

The sample extraction was carried out according to the method of Saikia et al. [7]. For core phenolic content (CPC) extraction, 0.1 g of the sample was dissolved in 1 mL of ethanol, acetic acid, and water (50:8:42) solution. The mixture was vortexed at room temperature for 1 min and filtered through a 0.45 µm Millipore filter. For the surface phenolic content (SPC) extraction, 0.1 g of the sample was dissolved in 1 mL of ethanol and methanol (1:1) solution. The mixture was vortexed at room temperature for 1 min and then filtered as mentioned above. The CPC and SPC were quantified by the Folin–Ciocalteu method [28]. Absorbance was measured at 765 nm with a spectrophotometer (Shimadzu UV–1240, Tokyo, Japan). The calibration curve was obtained with gallic acid at different concentrations (r^2^ = 0.9986). Results were expressed as milligrams of gallic acid equivalent per kilogram of powder (mg GAE/kg powder). The encapsulation efficiency was calculated according to the following Equation (1):

(1) $$Encapsulation\;efficiency\;\left(\%\right)=\frac{CPC-SPC}{CPC}\times \;100$$

where CPC is the phenolic content in the core of the microcapsule; SPC is the surface phenolic content.

### 2.5. Physical and Chemical Characterization of Microcapsules

Samples selected for further characterization were based on the highest encapsulation efficiency. For each wall material, the extract-to-wall material ratio with the highest efficiency was chosen.

#### 2.5.1. Particle Size and Zeta Potential

The average particle size and zeta potential of the microcapsules were determined by a Micromeritics Particulate Systems NanoPlus Zeta Analyzer (Micromeritics, Norcross, GA, USA) instrument. The capsules were dispersed in distilled water.

#### 2.5.2. Scanning Electron Microscopy

The surface morphology of the microcapsules was examined by a field emission scanning electron microscopy system (Quanta FEG 450, Oxford Instruments, Uedem, The Netherlands).

The samples were coated with a conductive (gold) layer using the SC7620 Mini Sputter Coater (Quorum Technologies, East Sussex, UK) and the imaging process was started. The images were captured with different magnifications of 1.5 KX, 3KX, 5KX, and 10KX using an acceleration voltage of 5 kV.

#### 2.5.3. Fourier Transform Infrared Spectroscopy Analysis

Infrared spectra of samples were obtained from wave number 400–4000 cm^−1^ using a Perkin Elmer Spectrometer (Perkin Elmer, Waltham, MA, USA) Spectrum Two Model by the ATR technique. Data were analyzed with Spectrum 10 Software.

#### 2.5.4. Differential Scanning Calorimetry Analysis

Differential Scanning Calorimetry (DSC) is a thermal analysis technique used to study the thermal behavior of materials. It measures how the heat flow to a sample changes as the temperature varies. DSC analysis was performed using Differential Scanning Calorimetry equipment (TA Instruments Q20, New Castle, DE, USA). Approximately 2.5 mg of the microcapsule samples was weighed. They were placed in an aluminum pan and heated from 25 °C to 350 °C at a scanning rate of 10 °C/min. In the analysis, nitrogen gas was set at a rate of 40 mL/min.

### 2.6. In Vitro Simulated Gastric and Intestinal Digestion Study of the Microcapsule

In vitro digestion of the encapsulated polyphenol content was examined by gastric and intestinal simulation, separately. The simulated gastric fluid (SGF) and simulated intestinal fluid (SIF) were prepared as given in U.S. Pharmacopeia [29].

For SGF preparation, sodium chloride (2.0 g) was mixed with pepsin from porcine stomach mucosa (3.2 g) (powder, ≥250 units/mg solid). Then, 7 mL of hydrochloric acid (37%) was added to the mixture, and the volume was adjusted to 1000 mL with water while maintaining a pH of 1.2. To simulate the digestion in the gastric fluid, 1.4 mL of SGF was added to 0.1 g of the encapsulated sample in a 10 mL test tube and incubated at 37 °C for 120 min at 80 rpm. The solution was filtered after cooling at room temperature and neutralized by adding 0.2 mol/L sodium hydroxide solution.

Similarly, SIF was prepared using monobasic potassium phosphate (6.8 g) dissolved in water (250 mL). To this solution, 0.2 N sodium hydroxide (77 mL) and pancreatin from porcine pancreas (10 g) (8 × USP specifications) were added. Then, the volume of the mixture was adjusted to 1000 mL, and a pH of 6.8 was maintained. To simulate the digestion in the intestinal fluid, 2.4 mL of SIF was added to a 10 mL test tube and incubated with 0.1 g of sample at 36.6 °C for 120 min with no shaking. The solution was filtered after cooling at room temperature, and enzyme activity was inhibited by decreasing the pH to 1.2 with 100 µL of 3 M hydrochloric acid added to 2 mL of filtrate. After 15 min, the solution was neutralized (pH 7.0) by adding 900 µL of 0.2 N sodium hydroxide. Lastly, both the samples of SGF and SIF were analyzed for apparent phenolic content by the Folin–Ciocalteu method. In addition, total phenolic content analysis was performed on the extracts prepared in pure water for 2 h without any digestion enzymes (at 37 °C), and the total phenolic content before digestion was calculated.

### 2.7. Antioxidant Capacity Analysis

Antioxidant capacities of microcapsules before and after in vitro digestion were determined with two different methods, which were DPPH scavenging activity and ABTS^•+^ scavenging activity assay. DPPH scavenging activity assay was performed using the method of Brand-Williams et al. [30] with some modifications. In a test tube, 100 µL of extract and 3 mL of DPPH solution (0.051 mmol/L) were added, and mixtures were incubated for 30 min at room temperature. Trolox solutions with different concentrations were used to construct a standard curve (r^2^ = 0.9983). Absorbance values were recorded at 517 nm using a spectrophotometer, and the results were expressed as milligram Trolox equivalent per kg powder. The ABTS assay was performed according to the method described by Re et al. [31]. ABTS^•+^ stock solution was prepared 16 h before use by reacting 2.45 mmol/L aqueous solution of K_2_O_8_S_2_ with 7mmol/L aqueous solution of ABTS^•+^. A water/ethanol mixture (1:1, *v*/*v*) was added to the stock solution until the absorbance value of the working solution was 0.70. Then, 50 µL of the extract was mixed with 3 mL of ABTS^•+^ working solution and incubated for 6 min at room temperature. The absorbance values were recorded at 734 nm by using a spectrophotometer, and the results were expressed as milligram Trolox equivalent per kg powder (r^2^ = 0.9994).

### 2.8. Statistical Analysis

The results of average particle size, zeta potential, encapsulation efficiency, phenolic content, and antioxidant capacity before and after in vitro digestion were statistically analyzed by ANOVA using the SPSS software (version 15 for windows, SPSS, Inc., Chicago, IL, USA). Duncan’s post hoc test was used to determine the differences between the groups (at *p* ≤ 0.05 significance level).

## 3. Results and Discussion

### 3.1. Encapsulation Efficiency

**Figure 1:**
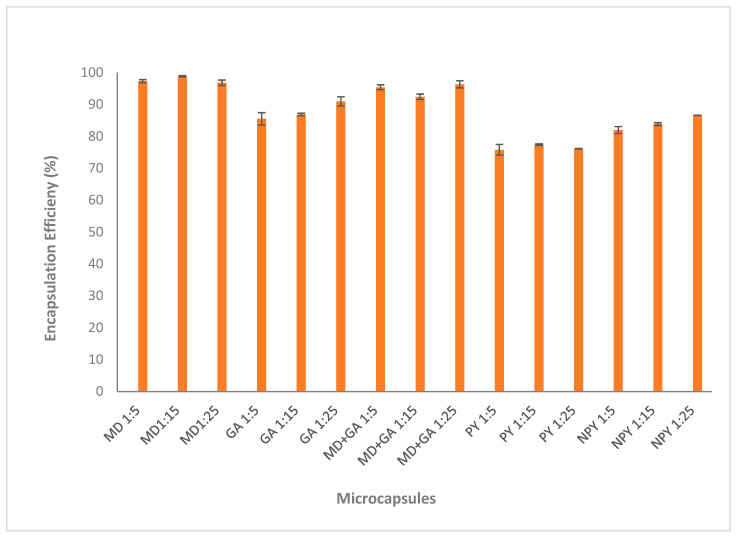
Encapsulation efficiency of all chokeberry microcapsules.

The encapsulation efficiency results of all chokeberry microcapsules are shown in Figure 1. In addition to this, the CPC and SPC values used in encapsulation efficiency calculations are included in Supplementary Material as Table S1. When maltodextrin was used alone as the coating material, the encapsulation efficiency remained statistically the same across all three concentrations and at its highest value. While the encapsulation efficiency was 90.97% in microcapsules coated using only gum arabic, the encapsulation efficiency increased to 96.31% when gum arabic was mixed with maltodextrin. These results are in agreement with the findings reported by Ballestroz et al. [2]. Researchers found that encapsulates with higher encapsulation efficiency in terms of phenolic content were obtained when 100% maltodextrin or gum arabic with maltodextrin was used as a coating material for the encapsulation of spent coffee grounds extracts by the freeze-drying method [2]. Maltodextrin (DE 10-12), which we used as the coating material in our study, has a higher molecular weight compared to gum arabic. Based on this, it can be inferred that phenolic substances form a tighter complex with the high-molecular-weight coating material. Therefore, higher EE values were obtained in MD-coated microcapsules [32]. In a previous study by Ćujić-Nikolić et al. [1], where chokeberry polyphenols were coated with maltodextrin, it was reported that a similarly high encapsulation efficiency was achieved.

When the extract-to-coating material ratio was 1:25, a higher encapsulation efficiency was achieved in samples coated with plasmolyzed and non-plasmolyzed yeast compared to other ratios. According to the results in Figure 1, the encapsulation efficiency was found to be lower in microcapsules using plasmolyzed yeast as the coating material. It is noticeable that plasmolysis treatment caused a decrease in the encapsulation efficiency of yeast cell microcapsules. Similar results were reported by Karaman [25], who encapsulated gallic acid into yeast cells. On the contrary, some researchers reported that plasmolysis treatment provides higher volume by removing intracellular content and so the encapsulation efficiency increased [23,33]. It is understood from this that the cell wall has an important place in the encapsulation process. In addition, the water content of the cell allows the yeast cell to exhibit hydrophilic properties and thus increase the diffusion of similar structures [34]. In the present study, due to the high hydrophilic structure of the phenolic components in the chokeberry extract, non-plasmolyzed yeast cell components showed higher encapsulation efficiency compared to plasmolyzed yeast. Young et al. [35] reported that more hydrophilic compounds can be encapsulated in higher concentrations due to the aqueous nature of the yeast cytoplasm. It has been suggested that the presence of intercellular cytoplasm and organelles in non-plasmolyzed yeast contributes to the binding of phenolic substances [35]. Based on our findings, the removal of cell components by plasmolysis did not consistently increase the amount of bound bioactive substances. Therefore, it is important to optimize plasmolysis treatment for yeast cells based on specific applications.

Finally, if we compare yeast cells and other coating materials in terms of encapsulation efficiency, microcapsules prepared with non-plasmolyzed yeast cells with an encapsulation efficiency of 86.58% can be seen as a competitor to gum arabic. In other studies, where phenolic substances were encapsulated with yeast, lower encapsulation efficiencies were observed. For example, Karaman et al. [25] found the encapsulation efficiency for yeast microcapsules containing gallic acid to be 37.9%, and Kurek et al. [26] found the encapsulation efficiency to be 53.88% for yeast microcapsules containing chokeberry anthocyanins. As a result, many parameters such as the bioactives in the extract used in encapsulation, the ratio of extract-to-wall material, and the encapsulation technique affect the encapsulation efficiency [19]. In the present study, chokeberry extract, which has a high polyphenolic substance content, was encapsulated with high efficiency by the freeze-drying method with a 1:25 extract–wall material ratio. It has been reported that higher encapsulation efficiencies can be achieved by using freeze-drying in the encapsulation of phenolic substances, which are heat-sensitive components [7].

### 3.2. Particle Size and Zeta Potential

The particle size of the produced encapsulates ranged from 0.87 to 2.62 µm (Table 1). The smallest particle size (0.87 ± 0.02 µm) was observed in the encapsulate obtained from gum arabic coating material. According to literature findings, particle sizes of encapsulates with gum arabic obtained by freeze-drying varied between 0.7 and 1.2 µm [36]. Encapsulates coated with maltodextrin were found to have a higher large particle size than encapsulates coated with gum arabic. Similar findings regarding the particle size of capsules coated with gum arabic and maltodextrin were reported by Tonon et al. [37]. It has been reported that maltodextrin with a low dextrose value can increase the particle size due to its larger molecular weight and molecular size [37,38]. It has been observed that capsules with smaller particle sizes are obtained when maltodextrin and gum arabic are used together for carrier mix, and a similar result was also obtained in our study [36]. The largest particle size was found in samples using plasmolyzed yeast as the coating material. The purpose of the plasmolysis process is to remove cytoplasmic components before encapsulation. It is an expected result that with this process, the molecular size of the yeast cells increases, and the particle size increases as the cell walls expand [39]. The higher particle size of the capsules allows more phenolic substances to be kept in the capsule [40]. The higher CPC values (Table S1) in MD 1:15, PY 1:25, and NPY 1:25 capsules with larger particle sizes confirmed this result. Thus, it was concluded that there is a correlation between CPC and particle size.

In the human mouth, colloidal particles smaller than 50 μm are typically not perceptible as individual entities, resulting in a “smooth” feeling. On the other hand, bigger particles are noticeable and, depending on their size, could result in a “rough” or “gritty” mouthfeel. In order to prevent microcapsules from affecting the food product’s mouthfeel or grittiness, it is crucial that they are smaller than 50 μm when utilized in food applications [41]. Our study indicates that all produced microcapsules have small particle sizes, which is not expected to negatively affect the mouthfeel.

Zeta potential is crucial for the stability of particles in dispersions, as it relates to aggregation phenomena and potential instability due to sedimentation. In our study, the zeta potentials of the capsules were determined to evaluate their physical stability. According to the results in Table 1, all encapsulates have negative values of zeta potential (−31.82/−38.23), indicating good electrostatic stabilization. Cujik-Nikoloviç et al. [13] reported a very similar zeta-potential value (−35 mV) for gum arabic-coated encapsulates. When the zeta potential is very low, the attractive forces overcome the repulsive forces, causing the particles to coalesce. Therefore, structures with high zeta potential are electrically stable compared to lower-zeta-potential ones [42].

### 3.3. Scanning Electron Microscopy Images

**Figure 2:**
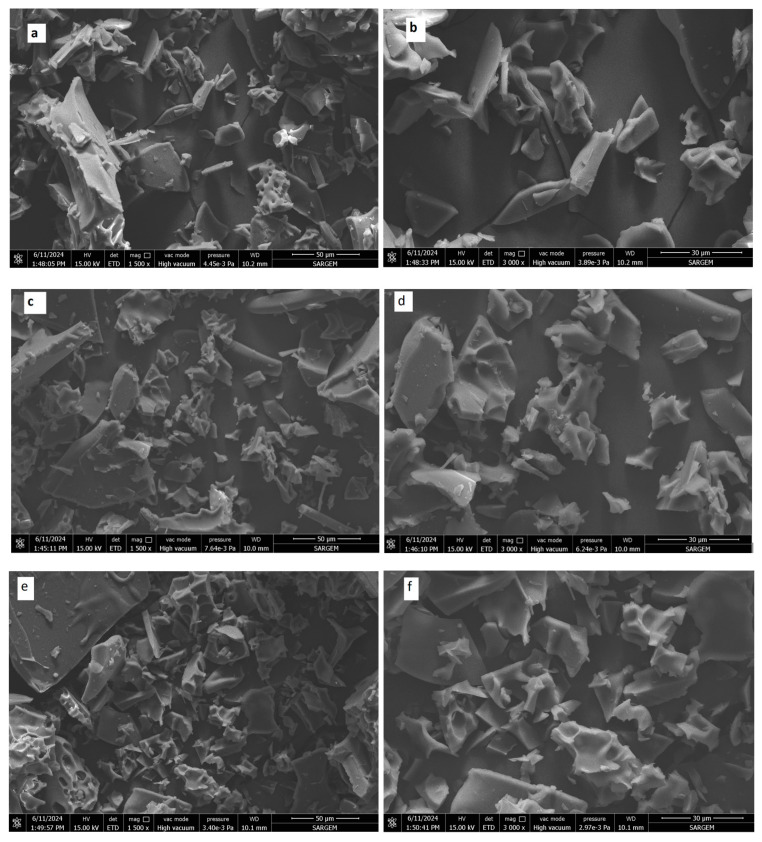

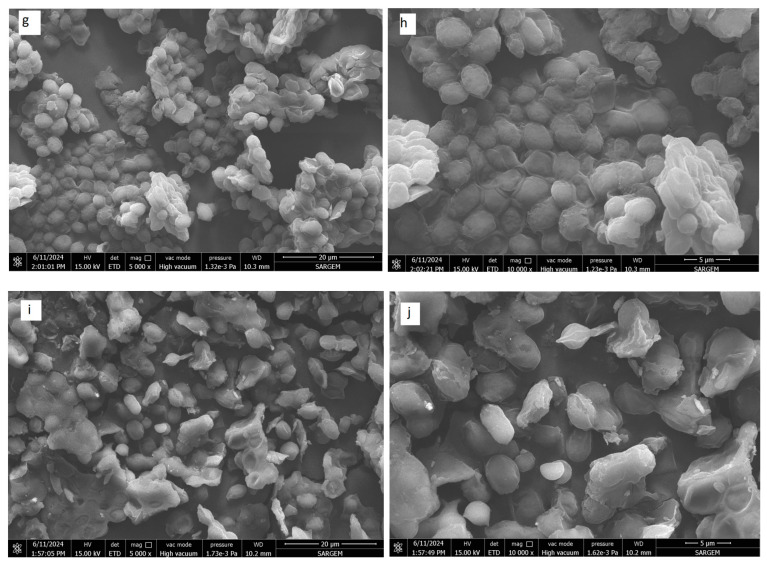
FESEM images of MD 1:15 (**a**,**b**), GA 1:25 (**c**,**d**), MD+GA 1:25 (**e**,**f**), PY 1:25 (**g**,**h**), NPY 1:25 (**i**,**j**). Magnification for (**a**,**c**,**e**) and (**b**,**d**,**f**) was 1.5KX and 3KX, respectively. Magnification for (**g**,**i**) and (**h**,**j**) was 5KX and 10KX, respectively.

SEM images of the capsules are illustrated in Figure 2. Significant differences in the surface morphology of samples are noticeable. Coating core material with maltodextrin and gum arabic by a freeze-drying process developed irregular shapes and caused a fragile structure in capsules. Likewise, Khazaei et al. [43] observed a similar amorphous glassy structure in saffron petal’s anthocyanin encapsulated with maltodextrin and gum arabic through freeze-drying. Freeze-drying modified the original morphology of maltodextrin and gum arabic by causing a more sawdust-like morphology [2]. The lack of forces in the freeze-drying process, which prevents breaking up the frozen liquid into droplets, may be the reason for the flat-like structure of GA1:25, MD1:15, and MD+GA1:25 samples [44].

The capsule surfaces had a characteristic spheroidal appearance when yeast was used as the coating material (Figure 2g–j). As is seen, cell agglomeration was evident in both yeast capsules (PY 1:25 and NPY 1:25). A similar observation was also confirmed by Kurek et al. [26], who explained agglomeration as a result of the freeze-drying process. Moreover, another reason for agglomeration may be due to the high β-glucan content of yeast samples [45,46]. Agglomeration observed in SEM images can be considered an optical illusion rather than a real phenomenon, possibly caused by the settling of yeast cells on the SEM sample holder surface (stub). When the particle size measured in dispersion was evaluated (Table 1), it was found to be similar to the size of a single yeast cell.

### 3.4. Fourier Transform Infrared Spectroscopy Results

**Figure 3:**
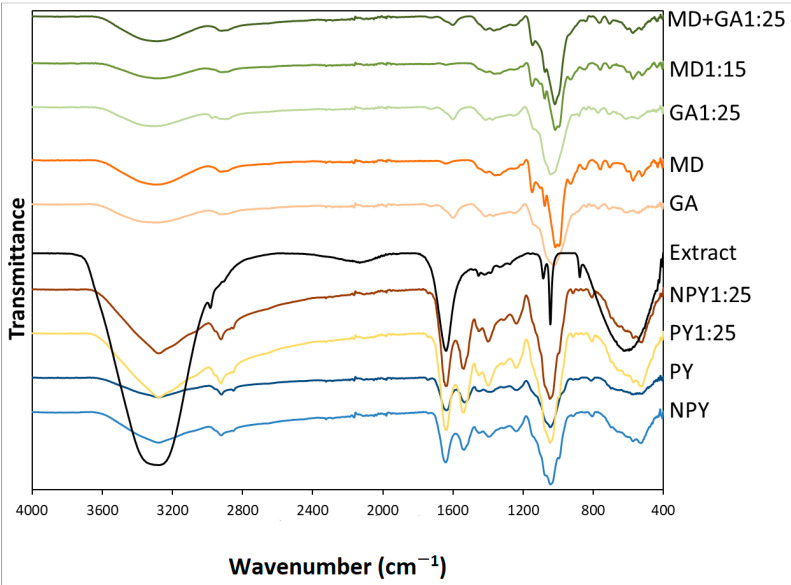
FT-IR spectrum of chokeberry extract, carriers, and chokeberry microcapsules.

Fourier transform infrared analysis was carried out to understand the differences in functional groups of samples after the encapsulation process. The spectrums of both matrices and encapsulated samples between 400 and 4000 cm^−1^ are shown in Figure 3. When the FTIR spectrums of MD and GA were examined, O–H stretching broad bands (hydroxyl group), C–H2 asymmetric stretching bands, and C–O stretching bands were observed at 3300, 2904, and 1000–1146 cm^−1^, respectively. The peaks at 1602 cm^−1^ and 836–772 cm^−1^ corresponded to the carboxylic acid group and O-H deformation vibrations of GA, respectively [2,47]. MD bands at 930, 850, 760, 703, and 570 cm^−1^ were associated with skeletal vibrations of the pyranoid ring, which agree with previous studies [48,49]. The absorption bands typical for pure MD and GA were also detected in encapsulated powders (GA1:25, MD1:15, MD+GA1:25); this showed that there were no new interactions between the carrier and chokeberry extract [2,13]. Silva et al. [50] also noted the similarity of the infrared spectra of the encapsulated grape pomace extract with maltodextrin. It can be concluded that the extract was successfully incorporated into the carrier materials during the freeze-drying process.

The FTIR spectra of empty yeast cells and their incorporation with chokeberry extract are also shown in Figure 3. The FTIR spectrum of non-plasmolyzed and plasmolyzed yeast cells showed an OH vibration peak of yeast polysaccharides (3268 cm^−1^), an asymmetric stretching peak of lipids (2919 cm^−1^), amide 1 and amide 2 peaks in proteins (1634 and 1532 cm^−1^), a dominant peak of β-glucan (1045 cm^−1^), and a peak of α-glucan (519 cm^−1^). These characteristic peaks belonging to yeast cells have also been documented in the literature [23,51]. The spectrum of chokeberry extract has a broad spectrum from 3700 to 2900 cm^−1^, which represents the O-H stretching vibration of carbohydrates and carboxylic acid. The reason for its high intensity was related to the water molecules. A characteristic band at 1640 cm^−1^ corresponds to the vibration of the C=O group of a glicon-pyranoside (galactoside, glucoside, and arabinoside) found in chokeberry extract [52]. The peaks at 1086 cm^−1^ and 1045 cm^−1^ indicated the presence of the stretching vibration of –C–O in cyclic ethers and the bending of the alcoholic group in phenols, respectively [53]. Although there were changes in the intensities between the microcapsules and yeast cells, the disappearance of the absorbance of extract indicated that polyphenols of chokeberry were encapsulated into the yeast cells [54].

### 3.5. Differential Scanning Calorimetry Results

**Figure 4:**
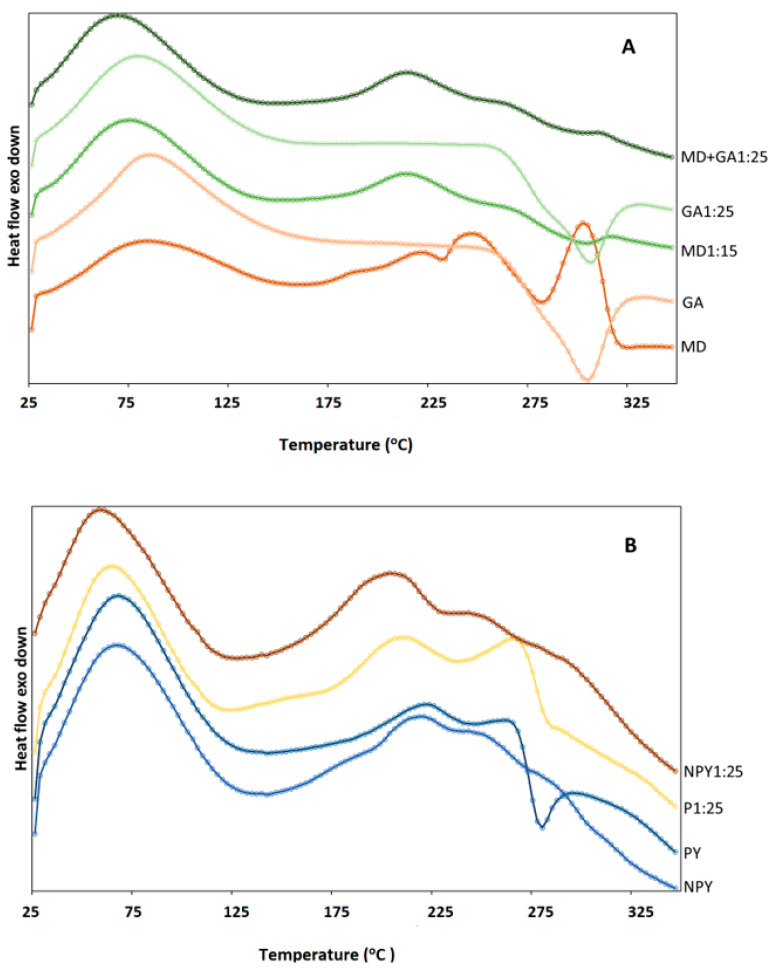
DSC spectrum of carriers and chokeberry microcapsules. (**A**) Maltodextrin and gum arabic; (**B**) yeast samples.

DSC analysis helps to determine a material’s physical and chemical properties by measuring its response to temperature changes. For example, determining a capsule’s thermal properties, such as its melting point or glass transition temperature, has a great impact on the stability and release rates of food supplements. Such characterization is critical for the effective release of active ingredients contained in capsules, especially in the pharmaceutical and food industries. Therefore, the data obtained with DSC spectra play a guiding role in food and drug design and development [55]. This study investigated the differences between the thermal behavior of microparticles and carriers (MD, GA, PY, and NPY). In Figure 4, a broad endothermic peak around 50–100 °C appeared in all samples and it was associated with the evaporation of loosely bound water [2,56]. These peaks in GA1:25, MD1:25, and MD+GA1:25 microparticles were insignificantly shifted to the lower temperatures. In yeast samples, the little endothermic peak at around 200 °C (220 °C for empty yeast cells and 208 °C for encapsulated ones) may be related to the phospholipid melting, which was also reported in the study of Ashkezary et al. [57]. Some changes were also observed in the DSC of the plasmolyzed cells, in which an exothermic peak at around 280 °C was detected, showing the thermal degradation of the loaded yeast cells. A similar result was also observed for the plasmolyzed microcapsules. No new peak between unloaded and loaded yeast samples indicated the successful encapsulation of anthocyanins in the yeast cell [51].

MD and GA carriers exhibited second exothermic events at 233 °C and 305 °C, respectively. A further temperature increase led to another peak at 285 °C in the MD sample. Melting and decomposition of these compounds could cause these exothermic processes above 250 °C [58]. While no shift in the exothermic peak was detected in GA-based microparticles, significant changes occurred in MD- and MD+GA-based microparticles. These results indicate that although all microparticles exhibited satisfactory thermal stability, which is a desirable property for the encapsulation process, optimal stability was achieved when GA was used as the sole wall material.

### 3.6. In Vitro Gastric and Intestinal Digestion of the Microcapsule Under Simulated Conditions

One of the important research topics of this study was to evaluate the changes in the total polyphenol content and antioxidant capacity of microcapsules under gastrointestinal conditions. The total polyphenol content of the microcapsules before and after digestion is presented in Table 2. After SGF treatment, the total phenolic content of MD, GA, MD+GA, NPY, and PY microcapsules decreased by 77.41%, 75.00%, 71.92%, 40.03%, and 36.55%, respectively. It can be said that the pepsin enzyme used in gastric digestion causes a decrease in phenolic compounds by increasing phenolic dissolution or interaction with specific phenolic compounds [59]. However, it is observed that the loss of phenolic compounds in the encapsulates coated with yeast is lower compared to other coating materials. As mentioned in the FTIR analysis results, it was possible to protect the phenolic compounds against pepsin digestive enzymes thanks to the interaction of structures such as β-glucan and α-glucan in the cell wall networks of yeast-coated capsules with chokeberry phenolic compounds [60]. Wu et al. [61] encapsulated blueberry extract with different coating materials such as gum arabic, maltodextrin, soy protein, and gelatin, and reported that the highest phenolic loss during digestion was in gum arabic (56.7%) and maltodextrin (64.7%). These findings are similar to our in vitro digestion results.

The DPPH and ABTS radical scavenging capacity of the microcapsules before and after digestion is presented in Table 3 and Table 4, respectively. Similarly to the total phenolic content, after SGF application, a decrease of 45.38–67.54% in DPPH scavenging activity and 32.95–57.61% in ABTS capacity were observed. Correa-Betanzo et al. [62] reported that a significant reduction (< 50%) in the DPPH scavenging activity of blueberry extract was observed after digestion. Considering that polyphenols exhibit strong antioxidant activity, this decrease is an expected result. After gastric digestion, the highest DPPH activities were observed in microcapsules coated with plasmolyzed (962.05 ± 20.68 mg Trolox/kg powder) and non-plasmolyzed yeast (989.00 ± 15.83 mg Trolox/kg powder), and there was no statistical difference between them (*p* < 0.05).

The reducing effect of SGF on the total phenolic content and antioxidant activity of the MA, GA, and MA + GA microcapsules was higher than the SIF treatment. The low pH value of the simulated gastric medium (pH 1.2) may be a reason for the greater reduction in phenolic and other antioxidant compounds. It is known that the degradation of anthocyanins occurs depending on pH during in vitro digestion [63]. After SIF treatment, the total phenolic content of MD, GA, MD+GA, NPY, and PY microcapsules decreased by 67.07%, 52.18%, 54.30%, 24.36%, and 22.45%, respectively. Similarly to gastric digestion, the pancreatin enzyme used in the SIF medium in intestinal digestion caused a decrease in phenolic compounds. In ABTS scavenging activity, the least loss (approximately 10%) was observed in non-plasmolyzed yeast samples after intestinal digestion. Still, the yeast used as a coating material efficiently protected phenolic and other antioxidant compounds in the in vitro intestinal tract. Jilani et al. [64] investigated the potential application of *Saccharomyces cerevisiae* as a carrier of phenolic compounds extracted from olive leaves. Researchers have verified that the degradation of phenolic compounds can be protected by yeast used as a coating material by determining the stability of oleuropein and hydroxytyrosol in the intestine during gastrointestinal digestion [64]. Similarly, results from the present study reveal that plasmolyzed or non-plasmolyzed yeast displayed a better protective effect on phenolic and other antioxidant compounds compared to maltodextrin and gum arabic after gastrointestinal digestion.

As a result, at the initial stage of the digestion, microcapsules typically dissolve in the stomach environment. Some polyphenols may begin to be released here; however, many are sensitive to acidic conditions and may undergo degradation before reaching significant absorption levels. The primary site for absorption of many nutrients, including polyphenols, is the small intestine. As the capsule moves into the duodenum (the first part of the small intestine), it encounters alkaline conditions. A portion of unabsorbed polyphenols continues to the colon, and they may still contribute beneficial effects through microbial metabolism [65]. Therefore, it is essential to investigate encapsulation materials that will not inhibit the release of phenolic compounds and prevent their loss in the stomach and intestine.

## 4. Conclusions

In this research, microencapsules prepared by freeze-drying exhibit high encapsulation efficiency of phenolic compounds (77.13–98.82%). Non-plasmolyzed yeast cell components showed higher encapsulation efficiency compared to plasmolyzed yeast. The largest particle size was observed in microcapsules coated with plasmolyzed yeast (2.62 µm). All encapsulates had a negative zeta potential value, indicating good electrostatic stabilization. Coating core material with maltodextrin and gam arabic by a freeze-drying process developed irregular shapes and caused a fragile structure in capsules, while the surface of yeast encapsulates had a characteristic spheroidal appearance. FTIR results showed a dominant peak of β-glucan and a peak of α-glucan for yeast encapsulates. After SGF and SIF digestion, the highest loss in phenolic compounds was observed in the samples coated with maltodextrin (77.41% and 67.07%, respectively), while the minimum loss was observed in the capsules coated with plasmolyzed yeast (36.55% and 22.45%, respectively). Furthermore, yeast microcapsules exhibited the lowest degradation in antioxidant activity following digestion. Our results showed that chokeberry microencapsulates coated with non-plasmolyzed yeast by the freeze-drying method could be a highly bioavailable dietary supplement and functional food product that provides additional health benefits. In addition to this, given their stability in digestive conditions and targeted particle size and thermal properties, these microencapsulates may also have potential in pharmaceutical formulations aimed at delivering different agents that require protection from degradation until they reach specific sites within the gastrointestinal tract.

## Figures and Tables

**Table 1:** Physical properties of chokeberry encapsulates produced using different carriers with the highest encapsulation efficiency.

Samples	Particle Size (µm)	Zeta Potential (mV)
MD 1:15	2.52 ± 0.02 ^c^	−38.23 ± 0.47 ^c^
GA 1:25	0.87 ± 0.02 ^b^	−37.70 ± 0.28 ^c^
MD+GA 1:25	1.09 ± 0.01 ^a^	−34.63 ± 0.56 ^b^
PY 1:25	2.62 ± 0.04 ^d^	−33.61 ± 0.69 ^ab^
NPY 1:25	2.47 ± 0.03 ^c^	−31.82 ± 0.16 ^a^

Different lowercase letters (a–d) for each column based on the Duncan test indicate a significant difference (*p* < 0.05) between the particle size or zeta potential of microcapsules.

**Table 2:** In vitro gastrointestinal digestion of total phenolic content of microcapsules in simulated gastric fluid (SGF) and simulated intestinal fluid (SIF).

	Total Phenolic Content (mg GAE/kg Powder)			Percentage Loss in Total Phenolic Content (%)	
	Before Digestion	SGF (After Gastric Digestion)	SIF (After Intestinal Digestion)	Percentage Loss (After Gastric Digestion)	Percentage Loss (After Intestinal Digestion)
MD 1:15	776.76 ± 7.34 ^bA^	173.23 ± 2.26 ^cB^	252.48 ± 4.81 ^dC^	77.41	67.07
GA 1:15	641.87 ± 15.08 ^cA^	160.52 ± 8.45 ^cB^	306.93 ± 7.10 ^cC^	75.00	52.18
MD+GA 1:25	625.87 ± 13.94 ^cA^	175.77± 3.19 ^cB^	286.03 ± 5.23 ^cC^	71.92	54.30
NPY 1:25	1075.20 ± 16.11 ^aA^	644.79 ± 15.51 ^aB^	813.23 ± 16.17 ^aC^	40.03	24.36
PY 1:25	756.09 ± 17.60 ^bA^	479.18 ± 13.22 ^bB^	586.32 ± 16.72 ^bC^	36.55	22.45

Different lowercase letters (a–d) for the first three columns based on the Duncan test indicate a significant difference (*p* < 0.05) between the total phenolic content of microcapsules while different uppercase letters (A–C) for the first three rows based on the Duncan test indicate a significant difference (*p* < 0.05) between the total phenolic content of microcapsules at different digestion phases.

**Table 3:** In vitro gastrointestinal digestion of DPPH scavenging activity of microcapsules in simulated gastric fluid (SGF) and simulated intestinal fluid (SIF).

	DPPH Scavenging Activity (mg Trolox/kg Powder)			Percentage Loss in DPPH Scavenging Activity (%)	
	Before Digestion	SGF (After Gastric Digestion)	SIF (After Intestinal Digestion)	Percentage Loss (After Gastric Digestion)	Percentage Loss (After Intestinal Digestion)
MD 1:15	2310.19 ± 51.70 ^a^	749.89 ± 13.20 ^a^	1108.36 ± 47.05 ^a^	67.54	52.02
GA 1:15	1310.19 ± 27.54 ^b^	491.77 ± 6.40 ^b^	676.49 ± 18.26 ^b^	62.47	48.37
MD+GA 1:25	834.72 ± 10.37 ^c^	305.08 ± 5.69 ^c^	385.51 ± 11.70 ^c^	63.45	53.82
NPY 1:25	1810.82 ± 31.60 ^d^	989.00 ± 15.83 ^d^	1507.49 ± 39.42 ^d^	45.38	16.75
PY 1:25	2144.15 ± 48.70 ^a^	962.05 ± 20.68 ^d^	1650.12 ± 62.73 ^d^	55.13	23.04

Different lowercase letters (a–d) for the first three columns based on the Duncan test indicate a significant difference (*p* < 0.05) between the antioxidant activity results of microcapsules.

**Table 4:** In vitro gastrointestinal digestion of ABTS scavenging activity of microcapsules in simulated gastric fluid (SGF) and simulated intestinal fluid (SIF).

	ABTS Scavenging Activity (mg Trolox/kg Powder)			Percentage Loss in ABTS Scavenging Activity (%)	
	Before Digestion	SGF (After Gastric Digestion)	SIF (After Intestinal Digestion)	Percentage Loss (After Gastric Digestion)	Percentage Loss (After Intestinal Digestion)
MD 1:15	596.60 ± 12.19 ^a^	252.91 ± 8.79 ^a^	347.31 ± 5.92 ^a^	57.61	41.79
GA 1:15	414.91 ± 11.67 ^b^	191.75 ± 8.81 ^b^	266.16 ± 9.32 ^b^	53.79	35.85
MD+GA 1:25	406.89 ± 6.67 ^b^	191.20 ± 6.92 ^b^	248.62 ± 4.98 ^b^	53.01	38.90
NPY 1:25	459.59 ± 6.09 ^c^	308.16 ± 15.21 ^c^	413.48 ± 6.21 ^c^	32.95	10.03
PY 1:25	524.40 ± 17.07 ^d^	342.20 ± 5.83 ^d^	439.12 ± 7.35 ^d^	34.75	16.26

Different lowercase letters (a–d) for the first three columns based on the Duncan test indicate a significant difference (*p* < 0.05) between the antioxidant activity results of microcapsules.

## Data Availability

The original contributions presented in the study are included in the article and Supplementary Material, further inquiries can be directed to the corresponding author.

## Supplementary Materials

The following supporting information can be downloaded at: https://www.mdpi.com/article/10.3390/foods14040625/s1, Table S1: CPC, SPC, and encapsulation efficiency of all chokeberry microcapsules.

## Abbreviations

The following abbreviations are used in this manuscript:

DSC	Differential Scanning Calorimetry
MD	Maltodextrin
GA	Gum arabic
MD+GA	Mixture of maltodextrin and gum arabic
PY	Plasmolyzed yeast
NPY	Non-plasmolyzed yeast
CPC	Core phenolic content
SPC	Surface phenolic content
GAE	Gallic acid equivalent
SGF	Simulated gastric fluid
SIF	Simulated intestinal fluid
SEM	Scanning electron microscopy
DPPH	2,2-Diphenyl-1-picrylhydrazyl
ABTS	2,2′-Azino-bis(3-ethylbenzothiazoline-6-sulfonic acid)

## References

[1] Ćujić-Nikolić N., Stanisavljević N., Šavikin K., Kalušević A., Nedović V., Samardžić J., Janković T. (2019). Chokeberry polyphenols preservation using spray drying: Effect of encapsulation using maltodextrin and skimmed milk on their recovery following in vitro digestion. J. Microencapsul..

[2] Ballesteros L.F., Ramirez M.J., Orrego C.E., Teixeira J.A., Mussatto S.I. (2017). Encapsulation of antioxidant phenolic compounds extracted from spent coffee grounds by freeze-drying and spray-drying using different coating materials. Food Chem..

[3] Gerasimov M.A., Perova I.B., Eller K.I., Akimov M.Y., Sukhanova A.M., Rodionova G.M., Ramenskaya G.V. (2023). Investigation of polyphenolic compounds in different varieties of black chokeberry *Aronia melanocarpa*. Molecules.

[4] d’Alessandro L.G., Kriaa K., Nikov I., Dimitrov K. (2012). Ultrasound assisted extraction of polyphenols from black chokeberry. Sep. Purif. Technol..

[5] Papoutsis K., Golding J.B., Vuong Q., Pristijono P., Stathopoulos C.E., Scarlett C.J., Bowyer M. (2018). Encapsulation of citrus by-product extracts by spray-drying and freeze-drying using combinations of maltodextrin with soybean protein and ι-carrageenan. Foods.

[6] Ćujić N., Bugarski B., Ibrić S., Pljevljakušić D., Šavikin K. (2016). Chokeberry (*Aronia melanocarpa* L.) extract loaded in alginate and alginate/inulin system. Ind. Crop Prod..

[7] Saikia S., Mahnot N.K., Mahanta C.L. (2015). Optimisation of phenolic extraction from Averrhoa carambola pomace by response surface methodology and its microencapsulation by spray and freeze drying. Food Chem..

[8] Grgić J., Šelo G., Planinić M., Tišma M., Bucić-Kojić A. (2020). Role of the encapsulation in bioavailability of phenolic compounds. Antioxidants.

[9] Buljeta I., Pichler A., Šimunović J., Kopjar M. (2022). Polysaccharides as carriers of polyphenols: Comparison of freeze-drying and spray-drying as encapsulation techniques. Molecules.

[10] Romero-González J., Ah-Hen K.S., Lemus-Mondaca R., Muñoz-Fariña O. (2020). Total phenolics, anthocyanin profile and antioxidant activity of maqui, *Aristotelia chilensis* (Mol.) Stuntz, berries extract in freeze-dried polysaccharides microcapsules. Food Chem..

[11] Timilsena Y.P., Haque M.A., Adhikari B. (2020). Encapsulation in the food industry: A brief historical overview to recent developments. Food Nutr. Sci..

[12] Ravichai K., Muangrat R. (2019). Effect of different coating materials on freeze-drying encapsulation of bioactive compounds from fermented tea leaf wastewater. J. Food Process. Preserv..

[13] Ćujić-Nikolić N., Stanisavljević N., Šavikin K., Kalušević A., Nedović V., Bigović D., Janković T. (2018). Application of gum Arabic in the production of spray-dried chokeberry polyphenols, microparticles characterisation and in vitro digestion method. Lek. Sirovine.

[14] Catalkaya G., Guldiken B., Capanoglu E. (2022). Encapsulation of anthocyanin-rich extract from black chokeberry (*Aronia melanocarpa*) pomace by spray drying using different coating materials. Food Funct..

[15] Jang Y., Koh E. (2024). Effect of encapsulation on stability of anthocyanins and chlorogenic acid isomers in aronia during in vitro digestion and their transformation in a model system. Food Chem..

[16] Do T.H., Kha C.T., Huynh P.P.T. (2019). Spray-drying microencapsulation of β-carotene by polysaccharide from yeast cell walls. J. Agric. Dev..

[17] Errenst C., Petermann M., Kilzer A. (2021). Encapsulation of limonene in yeast cells using the concentrated powder form technology. J. Supercrit. Fluids.

[18] Nguyen T.T., Phan-Thi H., Pham-Hoang B.N., Ho P.T., Tran T.T.T., Waché Y. (2018). Encapsulation of *Hibiscus sabdariffa* L. anthocyanins as natural colours in yeast. Food Res. Int..

[19] Dadkhodazade E., Khanniri E., Khorshidian N., Hosseini S.M., Mortazavian A.M., Moghaddas Kia E. (2021). Yeast cells for encapsulation of bioactive compounds in food products: A review. Biotechnol. Progr..

[20] Öztürk S., Cerit I., Mutlu S., Demirkol O. (2017). Enrichment of cookies with glutathione by inactive yeast cells (*Saccharomyces cerevisiae*): Physicochemical and functional properties. J. Cereal Sci..

[21] Sadeghi A., Ebrahimi M., Shahryari S., Kharazmi M.S., Jafari S.M. (2022). Food applications of probiotic yeasts; focusing on their techno-functional, postbiotic and protective capabilities. Trends Food Sci. Technol..

[22] Yiğit G.G., Cerit İ., Demirkol O. (2021). Oxidative stability of cocoa hazelnut cream enriched with inactive yeast cells. J. Food Process. Pres..

[23] Kavosi M., Mohammadi A., Shojaee-Aliabadi S., Khaksar R., Hosseini S.M. (2018). Characterization and oxidative stability of purslane seed oil microencapsulated in yeast cells biocapsules. J. Sci. Food Agric..

[24] Cerit İ. (2025). Evaluation of the effects of plasmolysis, solvent, and ultrasonication on encapsulation of lycopene in *Saccharomyces cerevisiae* cells. Food Bioprocess Technol..

[25] Karaman K. (2021). Fabrication of gallic acid loaded yeast (*Saccharomyces cerevisiae*) microcapsules: Effect of plasmolysis treatment and solvent type on bioactivity and release kinetics. LWT.

[26] Kurek M.A., Majek M., Onopiuk A., Szpicer A., Napiórkowska A., Samborska K. (2023). Encapsulation of anthocyanins from chokeberry (*Aronia melanocarpa*) with plazmolyzed yeast cells of different species. Food Bioprod. Process..

[27] Pham-Hoang B.N., Romero-Guido C., Phan-Thi H., Waché Y. (2018). Strategies to improve carotene entry into cells of *Yarrowia lipolytica* in a goal of encapsulation. J. Food Eng..

[28] Slinkard S., Singleton V.L. (1977). Total phenol analysis: Automation and comparison with manual methods. Am. J. Enol. Viticult..

[29] (2012). U.S. Pharmacopeia. Test Solutions. http://www.pharmacopeia.cn.

[30] Brand-Williams W., Cuvelier M.E., Berset C.L.W.T. (1995). Use of a free radical method to evaluate antioxidant activity. LWT.

[31] Re R., Pellegrini N., Proteggente A., Pannala A., Yang M., Rice-Evans C. (1999). Antioxidant activity applying an improved ABTS radical cation decolorization assay. Free Radic. Biol. Med..

[32] Laine P., Kylli P., Heinonen M., Jouppila K. (2008). Storage stability of microencapsulated cloudberry (*Rubus chamaemorus*) phenolics. J. Agric. Food Chem..

[33] Dadkhodazade E., Mohammadi A., Shojaee-Aliabadi S., Mortazavian A.M., Mirmoghtadaie L., Hosseini S.M. (2018). Yeast cell microcapsules as a novel carrier for cholecalciferol encapsulation: Development, characterization and release properties. Food Biophys..

[34] Normand V., Dardelle G., Bouquerand P.E., Nicolas L., Johnston D.J. (2005). Flavor encapsulation in yeasts: Limonene used as a model system for characterization of the release mechanism. J. Agric. Food Chem..

[35] Young S., Dea S., Nitin N. (2017). Vacuum facilitated infusion of bioactives into yeast microcarriers: Evaluation of a novel encapsulation approach. Food Res. Int..

[36] Šturm L., Črnivec I.G.O., Istenič K., Ota A., Megušar P., Slukan A., Ulrih N.P. (2019). Encapsulation of non-dewaxed propolis by freeze-drying and spray-drying using gum Arabic, maltodextrin and inulin as coating materials. Food Bioprod. Process..

[37] Tonon R.V., Brabet C., Hubinger M.D. (2010). Anthocyanin stability and antioxidant activity of spray-dried açai (*Euterpe oleracea* Mart.) juice produced with different carrier agents. Food Res. Int..

[38] Jamdar F., Ali Mortazavi S., Reza Saiedi Asl M., Sharifi A. (2021). Physicochemical properties and enzymatic activity of wheat germ extract microencapsulated with spray and freeze drying. Food Sci. Nutr..

[39] Pham-Hoang B.N., Romero-Guido C., Phan-Thi H., Waché Y. (2013). Encapsulation in a natural, preformed, multi-component and complex capsule: Yeast cells. Appl. Microbiol. Biotechnol..

[40] Esmaeilzadeh Kenari R., Razavi R. (2022). Phenolic profile and antioxidant activity of free/bound phenolic compounds of sesame and properties of encapsulated nanoparticles in different wall materials. Food Sci. Nutr..

[41] McClements D.J. (2018). Encapsulation, protection, and delivery of bioactive proteins and peptides using nanoparticle and microparticle systems: A review. Adv. Colloid Interface Sci..

[42] Seo E.J., Min S.G., Choi M.J. (2010). Release characteristics of freeze-dried eugenol encapsulated with β-cyclodextrin by molecular inclusion method. J. Microencapsul..

[43] Khazaei K.M., Jafari S.M., Ghorbani M., Kakhki A.H. (2014). Application of maltodextrin and gum Arabic in microencapsulation of saffron petal’s anthocyanins and evaluating their storage stability and color. Carbohyd. Polym..

[44] Chen C., Chi Y.J., Xu W. (2012). Comparisons on the functional properties and antioxidant activity of spray-dried and freeze-dried egg white protein hydrolysate. Food Bioprocess. Technol..

[45] Cichocki W., Czerniak A., Smarzyński K., Jeżowski P., Kmiecik D., Baranowska H.M., Kowalczewski P.Ł. (2022). Physicochemical and morphological study of the *Saccharomyces cerevisiae* cell-based microcapsules with novel cold-pressed oil blends. Appl. Sci..

[46] Sultana A., Miyamoto A., Hy Q.L., Tanaka Y., Fushimi Y., Yoshii H. (2017). Microencapsulation of flavors by spray drying using *Saccharomyces cerevisiae*. J. Food Eng..

[47] Sarabandi K., Jafari S.M., Mahoonak A.S., Mohammadi A. (2019). Application of gum Arabic and maltodextrin for encapsulation of eggplant peel extract as a natural antioxidant and color source. Int. J. Biol. Macromol..

[48] Kang Y.R., Lee Y.K., Kim Y.J., Chang Y.H. (2019). Characterization and storage stability of chlorophylls microencapsulated in different combination of gum Arabic and maltodextrin. Food Chem..

[49] Karaaslan M., Şengün F., Cansu Ü., Başyiğit B., Sağlam H., Karaaslan A. (2021). Gum arabic/maltodextrin microencapsulation confers peroxidation stability and antimicrobial ability to pepper seed oil. Food Chem..

[50] Silva J.T.D.P., Borges M.H., de Souza C.A.C., Fávaro-Trindade C.S., Sobral P.J.D.A., de Oliveira A.L., Martelli-Tosi M. (2024). Grape Pomace Rich-Phenolics and Anthocyanins Extract: Production by Pressurized Liquid Extraction in Intermittent Process and Encapsulation by Spray-Drying. Foods.

[51] Paramera E.I., Konteles S.J., Karathanos V.T. (2011). Microencapsulation of curcumin in cells of *Saccharomyces cerevisiae*. Food Chem..

[52] David I., Stefanut M.N., Cata A., Ienascu I., Pop R., Tanasie C. (2009). Study of anthocyanins from *Vaccinium myrtillus* L. frozen fruits. J. Agroaliment. Processes. Technol..

[53] Halász K., Csóka L. (2018). Black chokeberry (*Aronia melanocarpa*) pomace extract immobilized in chitosan for colorimetric pH indicator film application. Food Packag. Shelf Life.

[54] Shi G., Rao L., Yu H., Xiang H., Pen G., Long S., Yang C. (2007). Yeast-cell-based microencapsulation of chlorogenic acid as a water-soluble antioxidant. J. Food Eng..

[55] Levya Porras C., Cruz-Alcantar P., Espinosa-Solís V., Martínez-Guerra E., Piñón-Balderrama C.I., Compean Martínez I., Saavedra-Leos M.Z. (2019). Application of differential scanning calorimetry (DSC) and modulated differential scanning calorimetry (MDSC) in food and drug industries. Polymers.

[56] Magnaye M.J.F.A., Mopera L.E., Flores F.P. (2022). Effect of rice bran protein concentrate as wall material adjunct on selected physicochemical and release properties of microencapsulated β-carotene. Food Sci. Technol. Int..

[57] Ashkezary E.Z., Vazifedoost M., Nateghi L., Didar Z., Moslemi M. (2024). Characterization of encapsulated riboflavin in plasmolyzed and non-plasmolyzed *Saccharomyces cerevisiae* yeast cells. J. Food Meas. Charact..

[58] Kalušević A., Lević S., Čalija B., Pantić M., Belović M., Pavlović V., Nedović V. (2017). Microencapsulation of anthocyanin-rich black soybean coat extract by spray drying using maltodextrin, gum Arabic and skimmed milk powder. J. Microencapsul..

[59] Su D., Liu H., Zeng Q., Qi X., Yao X., Zhang J. (2017). Changes in the phenolic contents and antioxidant activities of citrus peels from different cultivars after in vitro digestion. Int. J. Food Sci. Technol..

[60] Fu D.W., Fu J.J., Li J.J., Tang Y., Shao Z.W., Zhou D.Y., Song L. (2022). Efficient encapsulation of curcumin into spent brewer’s yeast using a pH-driven method. Food Chem..

[61] Wu Y., Han Y., Tao Y., Li D., Xie G., Show P.L., Lee S.Y. (2020). In vitro gastrointestinal digestion and fecal fermentation reveal the effect of different encapsulation materials on the release, degradation and modulation of gut microbiota of blueberry anthocyanin extract. Food Res. Int..

[62] Correa-Betanzo J., Allen-Vercoe E., McDonald J., Schroeter K., Corredig M., Paliyath G. (2014). Stability and biological activity of wild blueberry (*Vaccinium angustifolium*) polyphenols during simulated in vitro gastrointestinal digestion. Food Chem..

[63] Huang Y., Zhou W. (2019). Microencapsulation of anthocyanins through two-step emulsification and release characteristics during in vitro digestion. Food Chem..

[64] Jilani H., Cilla A., Barberá R., Hamdi M. (2016). Improved bioaccessibility and antioxidant capacity of olive leaf (*Olea europaea* L.) polyphenols through biosorption on *Saccharomyces cerevisiae*. Ind. Crop Prod..

[65] Ali Redha A., Kodikara C., Cozzolino D. (2024). Does encapsulation improve the bioavailability of polyphenols in humans? A concise review based on in vivo human studies. Nutrients.

