# Supplementary material for: Utilization of Yeast Cells as Alternative Carriers in the Microencapsulation of Black Chokeberry (Aronia melanocarpa) Phenolic Extract
Source: Foods. 2025 Feb 13;14(4):625. doi: 10.3390/foods14040625 (PMC11854250; doi:10.3390/foods14040625)
Supplement: Supplementary file 1 [file foods-14-00625-s001.zip › foods-3474859-supplementary.pdf]

Utilization of yeast cells as alternative carrier in microencapsulation of black chokeberry (*Aronia melanocarpa*) phenolic extract

Özlem Aktürk Gümüşay [1], İnci Cerit [2] and Omca Demirkol [2]*

[1] Department of Gastronomy and Culinary Arts, Maltepe University, Maltepe, İstanbul, Turkey; ozlemgumusay@maltepe.edu.tr
[2] Department of Food Engineering, Sakarya University, Esentepe, Sakarya, Turkey; incicantik@sakarya.edu.tr (I.C); omcad@sakarya.edu.tr (O.D)
* Correspondence: omcad@sakarya.edu.tr

Table S1. CPC, SPC and encapsulation efficiency of all chokeberry microcapsules.

Samples	CPC (mg GAE/kg powder)	SPC (mg GAE/kg powder)	Encapsulation Efficiency (%)
MD 1:5	513.93 ± 14.61^{c}	13.97 ± 2.12^{fg}	97.28 ± 0.49^{a}
MD 1:15	167.6 ± 6.60^{g}	1.97 ± 0.24^{h}	98.82 ± 0.19^{a}
MD 1:25	81.60 ± 0.10i	2.63 ± 0.71^{h}	96.77 ± 0.87^{a}
GA 1:5	119.63 ± 10.14^{h}	17.47 ±3.77^{f}	85.48 ± 1.92^{de}
GA 1:15	43.97 ± 5.90^{jk}	5.8 ± 0.94^{gh}	86.83 ± 0.38^{d}
GA 1:25	38.47 ± 0.94^{k}	3.47 ± 0.47^{h}	90.97 ± 1.45^{c}
MD+GA 1:5	381.27 ± 16.18^{f}	17.80 ± 4.71^{f}	95.37 ± 0.77^{ab}
MD+GA 1:15	91.97 ± 0.71^{i}	6.97 ± 0.91^{gh}	92.42 ± 0.83^{bc}
MD+GA 1:25	65.97 ± 5.41^{ij}	2.46 ± 0.94^{h}	96.31 ± 1.13^{a}
PY 1:5	652.93 ± 6.60^{b}	157.97 ± 9.20^{a}	75.80 ± 1.65^{f}
PY 1:15	446.13 ± 18.86^{de}	100.80 ± 1.41^{c}	76.41 ± 0.22^{f}
PY 1:25	388.13 ± 11.89^{f}	92.630 ± 0.71^{c}	77.13 ± 0.07^{f}
NPY 1:5	762.60 ± 7.07^{a}	137.63 ± 9.66^{b}	81.96 ± 1.10^{e}
NPY 1:15	462.97 ± 12.49^{d}	74.80 ± 0.21^{d}	83.84 ± 0.44^{de}
NPY 1:25	420.63 ± 4.95^{e}	56.47 ± 0.94^{e}	86.58 ± 0.07^{d}

Different lowercase letters (a-k) for each column based on the Duncan test indicate a significant difference ($p<0.05$) between the CPC, SPC, or encapsulation efficiency of microcapsules.
